# Possible protective effects of resveratrol in hepatocellular carcinoma

**DOI:** 10.22038/IJBMS.2019.36821.8774

**Published:** 2020-01

**Authors:** Seda Cetinkaya Karabekir, Aydan Özgörgülü

**Affiliations:** 1 University of KTO Karatay, Faculty of Medicine, Department of Histology and Embryology, Konya, Turkey; 2 University of Necmettin Erbakan, Faculty of Meram Medicine, Department of Histology and Embryology, Konya, Turkey

**Keywords:** Apoptosis, Diethylnitrosamine, Hepatocellular carcinoma, In vivo, Resveratrol

## Abstract

**Objective(s):**

Resveratrol (RSV) is a naturally occurring plant polyphenol with cardioprotective, neuroprotective, immunoregulatory, and anticancer properties and is biologically effective against various diseases. This study aimed to investigate the chemopreventive effect of different doses of RSV against hepatocellular carcinoma (HCC) induced by diethylnitrosamine (DEN) in rats.

**Materials and Methods:**

The rats were randomly divided into six groups of seven rats each (n=42). The control group (group 1) was injected with saline, group 2 with dimethyl sulfoxide (DMSO), group 3 with DEN, group 4 with DEN and 50 mg/kg of RSV (DEN+RSV 50), group 5 with DEN and 75 mg/kg of RSV (DEN+RSV 75), and group 6 with DEN and 100 mg/kg of RSV (DEN+RSV 100). Pro-apoptotic Bax/anti-apoptotic Bcl-2 and tumor suppressor p53 markers were analyzed by immunostaining.

**Results:**

Superoxide dismutase, glutathione, and malondialdehyde concentrations were not statistically significant in DEN+RSV 100 group but were closest to the concentrations in control group. Liver function tests showed that enzyme activity (alanine aminotransferase, aspartate aminotransferase, alkaline phosphatase, and γ-glutamyl transferase) increased in DEN+RSV 50 and DEN+RSV 100 groups compared with control group but decreased in DEN+RSV 50 and DEN+RSV 100 groups compared with DEN group. Bax/Bcl-2 and p53 analysis showed a statistically significant increase in apoptotic cells in DEN+RSV 100 group.

**Conclusion:**

A 100 mg/kg dose of RSV may be a promising treatment for HCC.

## Introduction

Liver damage caused by various risk factors leads to an increase in the cellular cycle, resulting in regeneration. Inflammation is the primary result of the presence of any risk factor in the liver and leads to fibrosis and regeneration, two important indicators of cirrhosis; ~80% of Hepatocellular carcinoma (HCC) cases have their origin in cirrhosis (1).

 HCC is the sixth most common cancer and fourth leading cause of cancer associated mortality in the world (2). Three types of factors affect HCC development and progression: genetic, epigenetic, and environmental factors (3). 

Diethylnitrosamine (DEN) is one of the most important environmental carcinogenic substances. It is found in tobacco smoke, cosmetics, petrol, milk, processed foods such as meat products, fried fish, and alcoholic beverages (4). DEN metabolism produces reactive electrophilic intermediates that alter the structure of deoxyribonucleic acid (DNA) and act as initiating agents in two steps of hepatocarcinogenic studies, initiation and development (5). In addition, DEN causes an increase in the number of mutated liver cells and stimulates postnecrotic hepatocellular proliferation; therefore, it is accepted as a model in hepatocarcinogenic studies (6).

The application of plants in traditional cancer treatment is of great interest to researchers and has led to the exploration of phytocompounds from the natural biodiversity, predominantly from plant sources.

In addition, bioactive compounds that affect cancer pathways and direct cellular equilibrium toward cancer cell apoptosis might be a strong therapeutic strategy; phytocompacts are major functional food elements, and several of them have specific effects on key regulators of cancer cell apoptosis (7).

3,5,40-Trihy-droxystilbene (resveratrol [RSV]) occurs naturally in more than 70 plant species, such as *Polygonum cuspidatum*, mulberry (*Morus *sp.), table grapes, and red wine, and is a stilbene group compound containing phytoalexin. Many studies have reported that RSV offers a wide range of alternative protective and therapeutic options against different types of cancer (8, 9).

RSV shows chemopreventive and chemotherapeutic abilities in three stages of carcinogenesis: (i) formation, (ii) initiation, and (iii) progression. These abilities of RSV can be predominantly explained through cell cycle control and cancer cell apoptosis-inducing activities (10). RSV administration activates mitochondrial intrinsic apoptotic pathway components by releasing cytochrome c and Smac/diablo proteins, resulting in death of tumor cells (1).

The study result will be a positive outcome towards the further development of the resveratrol towards fight against HCC, which is reported to have limited therapeutic options.

## Materials and Methods

***Animal experiments***

In this study, we obtained 42 male albino Wistar rats weighing 180–200 g each from the laboratory of experimental animals (Konya, Turkey) (approval No. 26.02.2016-2016-006).

***Experimental protocol, HCC model creation, and treatment application***

RSV (R5010; Sigma-Aldrich Corporation, St. Louis, MO, USA) was dissolved in dimethyl sulfoxide (DMSO) (D0516; TCI America, Portland, OR, USA), and a DMSO control group was formed to monitor the effects of the DMSO dose. Control group received a single dose of 0.9% NaCl solution intraperitoneally (IP) for 56 days. 

DMSO was administered IP in a 1:1 ratio for 7 days. DEN and DEN+RSV 100 groups received DEN IP at a dose of 100 mg/kg once a week for 7 weeks to reduce the risk of mortality; DEN administration resulted in liver damage.

While the HCC rat model was being formed, we conducted a preliminary study by referring to the literature for dose adjustment and performed histopathological examinations of liver tissues obtained from male albino Wistar rats. The experiment was begun after we observed HCC development. The end of 7 weeks, 24 hr after receiving the latest medication, RSV in DMSO solvent were administered via IP for 7 days at a dose of 50, 75, and 100 mg/kg/day in DEN treated rats, respectively (12).

***Measurement of body weight***

The rats were weighed 1 week before treatment and once a week during treatment. We can observe a decrease in the weight of rats when forming cancer. Therefore the weights of rats were noted every week.

***Preparation of tissues and blood samples***

After 56 days, the rats were anesthesized using a ketamine /xylazine 50/10 mg/kg injection. Blood samples were drawn from their hearts into heparin tubes. 

Plasma was obtained by centrifugation to measure the parameters of superoxide dismutase (SOD), glutathione (GSH), malondialdehyde (MDA), aspartate aminotransferase (AST), alanine aminotransferase (ALT), alkaline phosphatase (ALP) and γ-glutamyl transferase (GGT) from blood samples plasma of samples were stored in the freezer at-20 ^°^C until measurements were made (13).

***Biochemistry***

At the end of the experiment, blood plasma were analyzed in Teknik Kimya Medikal (Konya Turkey). Plasma was obtained from the stored blood samples by centrifugation, and the concentrations of SOD, GSH, MDA, ALT, AST, ALP, and GGT were measured.

***Histopathology***

The abdominal cavities of the rats were opened, and liver tissues were macroscopically examined. Then tissues were stored in a 10% neutral formaldehyde solution for 24-48 hr.

For microscopic examination, 5-µm-thick paraffin block sections were taken as normal slides for routine hematoxylin–eosin (H&E) staining and poly-L-lysine-coated slides were used for immunohistochemistry staining. The sections were examined under a BX-051 research microscope (Olympus Corporation, Tokyo, Japan) and photographed.

***Immunohistochemistry***

Pro-apoptotic Bax/anti-apoptotic Bcl-2 and tumor suppressor p53 expression in cells was determined on the basis of the intensity of staining. The staining intensity was graded as follows: 0, no staining; +1, light staining; +2, moderate staining; and +3, strong staining. Then, HE-stained sections and sections whose Bax/Bcl-2 and p53 markers were analyzed by immunohistochemistry staining were examined and the possible protective effects of RSV doses (cancer cell apoptosis) in HCC evaluated.

***Evaluation of fibrosis***

All liver specimens were fixed in 10% neutral formaldehyde solution, embedded in paraffin and routinely processed for histological analysis. Fibrosis scoring was performed according to the Metavir scoring system. “F” refers to the extent of fibrosis and may vary from F0 to F4 (F0=no fibrosis, F1=portal fibrosis without septa, F2=portal fibrosis with rare septa, F3=numerous septa without cirrhosis, and F4=cirrhosis) (14).

***Statistical analysis***

The recorded weights of the rats were evaluated using the paired sample t-test. One-way anlaysis of variance (ANOVA) and Duncan’s test were applied to SOD, GSH, MDA, ALT, AST, ALP, and GGT values. The Kruskal-Wallis test was used to measure differences in fibrosis between groups.

## Results

***Body weight findings***

Table 1 shows the results of the paired sample *t*-test for evaluating body weight measurements of the rats.

As can be seen from Table 1, at the end of the experiment, the body weights of DEN, DEN+RSV 50, DEN+RSV 75 groups, and DEN+RSV 100 group were significantly low compared with control group and DMSO group. The different letters show statistical significance.

***Biochemical findings***

Tables 2 and 3 show the results of the comparison of antioxidant capacities and plasma biochemical parameters, respectively, of the six groups. The different letters show statistical significance (*P*<0.05).

The SOD, GSH, and MDA concentrations in DEN+RSV 100 group were closest to those in control group, although the concentrations were not statistically significant.

Liver function tests showed that enzyme activity (ALT, AST, ALP, and GGT) increased in DEN+RSV 50, DEN+RSV 100 groups compared with control group but decreased in DEN+RSV 50, DEN+ RSV 100 groups compared with DEN group.

***Histopathological findings***

**Figure 1:**
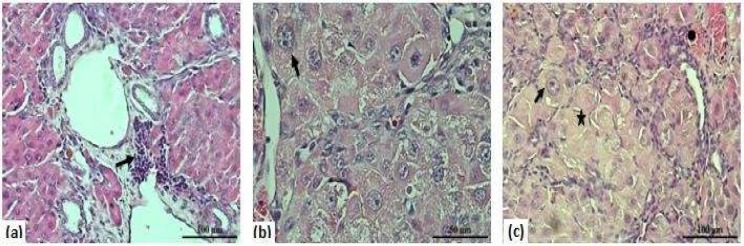
Representative photomicrographs pathological formations of the liver in rats (a): ( Lymphocyte infiltration (arrow)-100X, (b): Atypical cell (arrow)-400X, (c): Focal necrotic area (*), atypical cell (arrow), congestion (+) -100X (H&E Staining)

Microscopic examination of liver tissues showed bile duct proliferation, sinusoidal dilatation and degeneration, vascular congestion, lymphocyte infiltration in portal areas, fibrosis in liver nodules, dysplastic changes in the fibrosis parenchyma, necrosis, cellular atypia, and large tumor foci in DEN, DEN+RSV 100 groups (Figure 1).

In control group, histological sections of the liver demonstrated normal organization of liver lobules consisting of one-to two-cell-thick liver cords spreading from the central vein to the lobular environment. DMSO group showed moderate venous and sinusoidal occlusion of liver tissues, with no significant difference compared with control group.

Kruscal Wallis test (*P*-value=0.000) showed differences in fibrosis between groups. There was no significant difference between the DEN+RSV 50 (*P*-value =0.317) and DEN+RSV 75 (*P*-value = 0.014) groups when compared to the DEN group, according to the fibrosis score of the experimental groups. However, lymphocyte infiltration, fibrosis and nodular structures decreased in DEN+RSV 75 group. There was significant difference in DEN+RSV 100 group (*P*-value=0.004) (Table 4). 

**Figure 2:**
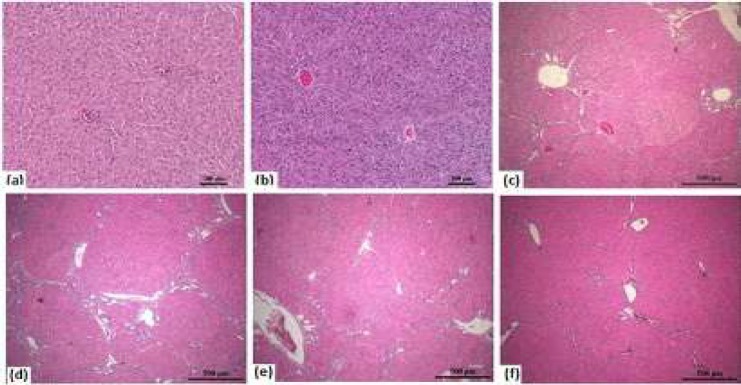
H&E staining according to groups of liver tissue -100X. Representative photomicrographs fibrosis formations of the liver in rats (Figure 2 c-f) (a) Control group (b) DMSO positive control, (c) DEN group, (d) DEN+RSV 50 groups, (e) DEN+RSV 75 groups, (f) DEN+RSV 100 group

Control group liver sections showed sinusoids located between the vena centralis and the hepatocyte cords, which showed normal radial alignment (Figure 2a). DMSO group liver sections showed no differences compared with control group (Figure 2c). Figure 2d–f shows fibrosis that separates or tends to separate the liver into nodules and dysplastic changes in the parenchyma and atypical cells.

***Immunohistochemistry findings***

DEN+RSV 50 and DEN+RSV 75 groups showed a decrease in Bcl-2 expression compared with DEN group, although the decrease was not statistically significant. However, the decrease in Bcl-2 expression was significant in DEN+RSV 100. Compared with DEN group, Bax and p53 expression levels in DEN+RSV 50 and DEN+RSV 100 groups increased with increasing RSV doses. We observed no significant difference in Bcl-2 expression level between DMSO *P*-value= 0.655), DEN+RSV 75 (*P*-value=0.055) and DEN+RSV 100 groups (*P*-value=0.866). However, we found a statistically significant difference between DEN group, (*P*-value=0.009) and DEN+RSV 50 group (*P*-value=0.027) and between control group and DEN (*P*-value=0.006), DEN+RSV 50, (*P*-value=0.016), and DEN+RSV 75 (*P*-value=0.031) groups. However, there was no significant difference between control group and DEN+RSV 100 group (*P*-value=0.513): the Bcl- 2 expression level in DEN+RSV 100 group was closest to that in control group (Table 5).

**Figure 3:**
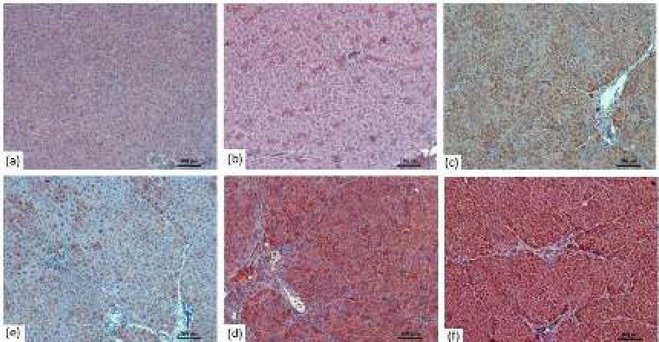
Immunohistochemical image of Bax expression relative to groups of liver tissue (100X) a) Control group (b) DMSO positive control, (c) DEN group, (d) DEN+ RSV 50 group, (e) DEN+ RSV 75 group, (f) DEN+ RSV 100 group

**Figure 4:**
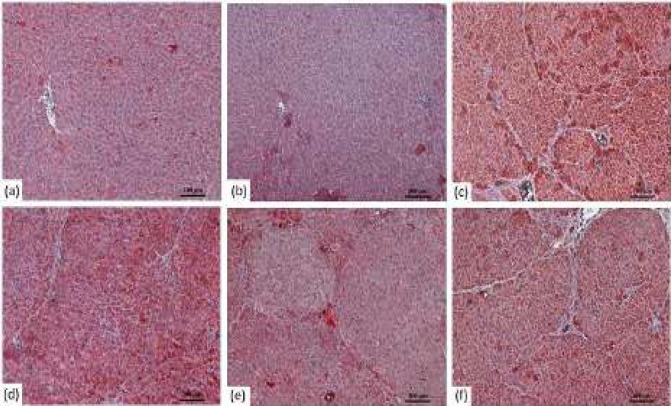
Immunohistochemical image of Bcl-2 expression relative to groups of liver tissue in rats (100X) (a) Control group (b) DMSO positive control, (c) DEN group, (d) DEN+ RSV 50 group, (e) DEN+ RSV 75 group, (f) DEN+ RSV 100 group

**Figure 5.**
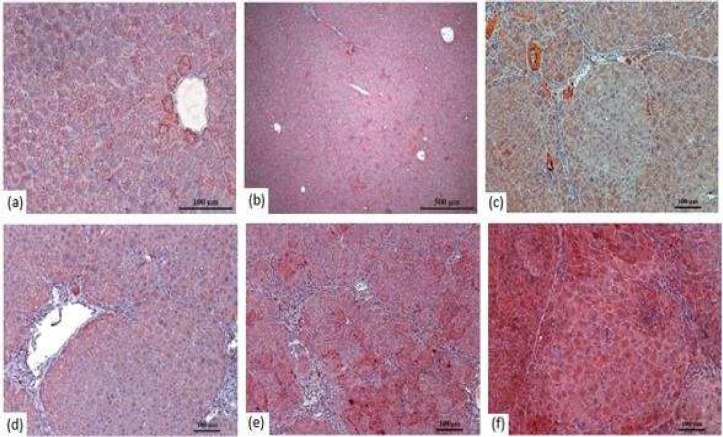
Immunohistochemical image of p53 expression relative to groups of liver tissue in rats (100X) (a) Control group (b) DMSO positive control, (c) DEN group, (d) DEN+ RSV 50 group, (e) DEN+ RSV 75 group, (f) DEN+ RSV 100 group

Our study showed up-regulation of Bax and down-regulation of Bcl-2 in HCC rats (Figures 3-5). The Bax expression in DEN+RSV 100 group showed a significant increase (Table 5), whereas the p53 expression level showed a significant increase in DEN+RSV 75 and DEN+RSV 100 groups (Table 5)

## Discussion

Nitrosamines are carcinogenic compounds, and DEN is one such nitrosamine that causes HCC (15). DEN is metabolized to reactive electrophilic intermediates that alter the DNA structure. DEN also increases the number of mutated liver cells and stimulates postpartum hepatocellular proliferation (6).Most of the metabolic events are caused by HCC development due to DEN, and the liver is the most affected organ.

The goal of using various natural and/or synthetic substances in cancer treatment is two fold: to inhibit cancer cell proliferation and to induce cancer cell apoptosis (16).

In this study, because of its RSV’s antioxidant and anticarcinogenic properties, we used different doses of RSV in an HCC model of albino male Wistar rats and examined the possible protective effects of those RSV doses by analysis of Bax/Bcl-2 and p53 expression and biochemical parameters.

Recent studies have reported that RSV might have protective effects in different types of cancer (16). Several studies have also shown that RSV might show cytotoxicity and cancer cell apoptosis-inducing properties in different cancer cell lines. In addition, RSV might even decrease the rate of proliferation of cancer cells (17).

We macroscopically observed nodules in the liver in DEN-induced HCC groups. In DEN-induced group, sinusoidal dilatation and degeneration, vascular congestion, lymphocyte infiltration in portal areas, disruption of the lobules, dysplastic changes in the fibrosis parenchyma, necrosis, cellular atypia and large tumor foci were observed.

This result was consistent with the report by Bishayee and Dhir (2009) (12). Kadasa *et al.* (2015) reported that histological sections of rats in the control group showed normal hepatic lobe organization, normal hepatocytes, and sinusoidal architecture (18). In DEN-induced HCC rats (a 200 mg/kg single dose of DEN), researchers observed focal replacement of portal areas, mostly collapsed membranes, an inadequate basophilic cytoplasm, large vesicular nuclei, a large amount of mitosis, and large fibrous structures with colichio adenocarcinoma islets (18). Khan *et al.* (2017) used DEN to induce HCC in male albino Wistar rats. They dissolved DEN in corn oil and administered it through intragastric gavage in two doses (180 mg/kg body weight) at 15 day intervals. After DEN administration, they found that the liver’s characteristic histological features (i.e., a hexagonal lobular structure with radially regulated hepatocytes, the central vein, and the peripheral portal triad) were scattered or damaged. This result demonstrated the chain of events starting with oxidative stress and moving on to membrane damage, inflammatory cell infiltration, cellular atypia, and, consequently, HCC development (19).

Bishayee and Dhir (2009) reported that the number of nodules and the density of nodules significantly decreased in groups treated with three doses of RSV (50, 100, and 300 mg/kg) compared with the DEN-induced HCC group; however, the result was more prominent in groups administered 100 and 300 mg/kg of RSV (*P<*0.001) (12).

In our study, weights of all rats in experimental groups were recorded before application and on a weekly basis during the application. According to recorded body weight results, the body weights of DEN induced rats decreased significantly compared to the control group.

DEN-induced HCC rats show decreased appetite; therefore, decreased food intake and weight loss might be indirect indicators of decreased liver function after HCC development. Studies have shown that HCC results in rapid and progressive loss of body weight, especially skeletal muscle and adipose tissue loss, with relative preservation of visceral proteins. This reduction can be explained primarily by accelerated protein catabolism (20).

Most hepatotoxic chemicals damage liver cells by increasing lipid peroxidation and causing other oxidative damage. Through its enzymatic and nonenzymatic components, the antioxidative defense system can clear up reactive oxygen specides, which play an important role in lipid peroxidation initiation. Glutathione peroxidase (GSH-Px) and SOD are two important antioxidant enzymes that eliminate free radicals from the body; GSH “cleans” electrophilic parts produced by toxic chemicals and conjugates them into less toxic products (21).

Oxidative stress, characterized by an increase in active oxygen groups caused by nitrosamines, increases the MDA concentration, indicating lipid peroxidation, changes in the GSH concentration, and antioxidant enzyme (GSH-Px and catalase [CAT]) activity. The GSH concentration decreases in the blood, liver, and kidneys because of nitrosamines, but this decrease is not statistically significant (22).

In our study, in terms of SOD, GSH and MDA levels, DEN + Res 100 group is the closest dose group to the control group among resveratrol-treated groups, although not statistically significant.

Granado-Serrano *et al.* (2009) showed that the GSH concentration decreases significantly in the DEN-induced HCC group compared with the control group (23). Other studies have shown a significant decrease in SOD, CAT, GSH-Px, and glutathine reductase activity and GSH concentration and a significant increase in MDA concentration in DEN-induced HCC groups (24, 25).

ALT, AST, ALP, GGT, and total bilirubin activity represent liver function, and increased concentrations of these enzymes are sensitive indicators of hepatic damage (26). AST and ALT are directly related to the conversion of amino acids to ketoacids and the increase in HCC-conducive conditions (21). Furthermore, a significant increase in GGT concentrations in rat plasma is because of the passage of GGT from the plasma membrane into the blood, resulting in cell membrane damage due to carcinogenesis (27).

In our study, there was an increase in liver enzyme levels (ALT, AST, ALP, GGT) in the resveratrol groups but there was a decrease compared to the DEN group. This decrease is not statistically significant. However, the tendency of resveratrol to decrease the elevated liver enzyme levels caused by DEN suggests that it may have a protective effect on liver function. 

Chen *et al.* (2012) showed that an increase in plasma AST, ALT, ALP, GGT, and α-l-fucosidase activity might cause hepatic dysfunction (21).

ALP is closely related to the lipid membrane in the canalicular region. Elevated ALP concentration reflects pathological changes in bile flow; that is, any intervention in extrahepatic or intrahepatic bile flow leads to increased plasma ALP concentration (28). Al-Rejaie *et al.* (2009) reported that compared with the control group, DEN administration 8 weeks after their experiment resulted in an increase in plasma ALT, GGT, and ALP concentrations of of 316%, 152%, and 219%, respectively (29). 

The tendency of RSV to decrease elevated liver enzyme concentrations due to DEN suggests that RSV may have protective effects on liver function. These data also indicate that RSV has an inhibitory effect on DEN-induced HCC development.

Krishnan *et al.* (2017) used immunohistochemical staining to examine the expression of apoptotic and anti-apoptotic-related proteins (Bcl-2, cyclooxygenase-2, and inducible nitric oxide synthase) in the hepatic region. Interestingly, studies have also detected the immunohistochemical staining of these three proteins in the cytoplasm of hepatocytes of all animals treated with DEN (30).

Ou *et al.* (2014) reported that RSV inhibits expression of Bcl-2 and increases expression of Bax and p53. Therefore, they suggested that RSV induces cancer cell apoptotis via a caspase- and p53-dependent pathway. As we observed in this study, p53 pathway activation upon RSV administration has also been previously reported (31).

Rajasekaran *et al.* (2011) reported an increase in p53 expression by RSV treatment and activation of the p53-dependent apoptotic pathway. In addition, a significant decrease in p53 expression in HCC rats confirms the anti-apoptotic and oncogenic mechanisms associated with DEN-induced HCC development in rats (32). Our findings of up-regulation of Bax and down- regulation of Bcl-2 in HCC rats were compatibile with other studies.

**Table 1:** Body weight measurements of rats evaluated by the paired sample *t*-test

	**Start of experiment** **Mean + SD**	**End of experiment** **Mean + SD**
**CONTROL**	235.2 ± 20.09**a, b**	409 ± 36.07 **a**
**DMSO**	239.25 ± 9.03 **a, b**	439.25 ± 40.77 **a**
**DEN**	242.2 ± 12.09 **a, b**	339.8 ± 24.64 **b**
**DEN + RSV 50**	231.6 ± 10.26**a, b**	291 ± 42.6 **b**
**DEN + RSV 75**	224.8 ± 17.54 **a**	313.2 ± 47,84 **b**
**DEN + RSV 100**	248 ± 5,47 **b**	325 ± 37.68 **b**

DMSO: dimethylsulfoxide; DEN: diethylnitrosamine; RSV: resveratrol. (a, b and c represent similarities and differences in statistics)

**Table 2.** Comparison of antioxidant parameters of liver tissue of rats by one-way ANOVA and Duncan’s test

	**SOD** **Mean + SD**	**MDA** **Mean + SD**	**GSH** **Mean + SD**
**CONTROL**	0.4 ± 0.13**a**	0.21 ± 0.03**a**	0.25 ± 0.02 **a**
**DMSO**	0.35 ± 0.03**a,c**	0.22 ± 0.01**a, b**	0.23 ± 0.009 **a,b**
**DEN**	0.25 ± 0.04**b**	0.28 ± 0.009 **c**	0.18 ± 0.04 **c**
**DEN + RSV 50**	0.28 ± 0.01**b,c**	0.25 ± 0.03**b,** **c**	0.2 ± 0.03 **b,c**
**DEN + RSV 75**	0.3 ± 0.04**b,c**	0.24 ± 0.03**a, b, c**	0.19 ± 0.03 **b,c**
**DEN + RSV 100**	0.32 ± 0.03**a,b,c**	0.24 ± 0.02**a, b**	0.22 ± 0.02 **a,b**

DMSO: dimethyl sulfoxide; DEN: diethylnitrosamine; RSV: resveratrol; SOD: superoxide dismutase; GSH: glutathione; MDA: malondialdehyde. (a, b and c represent similarities and differences in statistics)

**Table 3:** Comparison of plasma marker enzymes of hepatic damage in all groups of rats by one-way ANOVA and Duncan’s test

	**AST** **Mean + SD**	**ALT** **Mean+SD**	**ALP** **Mean+SD**	**GGT** **Mean+SD**
**CONTROL**	108.6 ± 21,07**a**	40 ± 3.3 **a**	222.4 ± 33.01 **a**	2.4 ± 0.24 **a**
**DMSO**	103.5 ± 11.2**a**	47.5 ± 5.26 **a**	322 ± 62.96 **a**	2.25 ± 0.25 **a**
**DEN**	252.8 ± 35.08**b**	187 ± 25.81 **b**	721.2 ± 106.26 **b**	16.2 ± 4.5 **b**
**DEN + RSV 50**	215.14 ± 35.74 **b**	130.14 ± 13.11**b**	683.42 ± 112.01 **b**	9.28 ± 2.27**a,b**
**DEN + RSV 75**	289.33 ± 42.71**b**	183.66 ± 32.21**b**	678.83 ± 35.02 **b**	10.16 ± 2.28 **a,b**
**DEN + RSV 100**	208.83 ± 23.44**b**	149.5 ± 21.88 **b**	615.5 ± 62.72 **b**	10.16 ± 2.49**a,b**

DMSO: dimethyl sulfoxide; DEN: diethylnitrosamine; RSV: resveratrol; AST: aspartate aminotransferase; ALT: alanine aminotransferase; ALP: alkaline phosphatase; GGT: γ- glutamyl transferase. (a, b and c represent similarities and differences in statistics)

**Table 4:** Fibrosis scoring of the liver tissue in the all groups of rats by the Kruskal–Wallis test

Groups		*P*-value
DMSO	CONTROL	0.371
DMSO	DEN	0.005
DMSO	DEN + RSV 50	0.007
DMSO	DEN + RSV 75	0.007
DMSO	DEN + RSV 100	0.007
CONTROL	DEN	0.004
CONTROL	DEN + RSV 50	0.005
CONTROL	DEN + RSV 75	0.005
CONTROL	DEN + RSV 100	0.015
DEN	DEN + RSV 50	0.317
DEN	DEN + RSV 75	0.014
DEN	DEN + RSV 100	0.004
DEN + RSV 50	DEN + RSV 75	0.072
DEN + RSV 50	DEN + RSV 100	0.005
DEN + RSV 75	DEN + RSV 100	0.005

DMSO: dimethyl sulfoxide; DEN: diethylnitrosamine; RSV: resveratrol

**Table 5:** Changes in Bcl-2, Bax and p53 expression between groups

Groups		*P*-value Bcl-2	*P*-value Bax	*P*-value p53
DMSO	CONTROL	0.655	0.655	0.866
DMSO	DEN	0.009	0.18	0.655
DMSO	DEN + RSV 50	0.027	0.107	0.273
DMSO	DEN + RSV 75	0.055	0.023	0.009
DMSO	DEN + RSV 100	0.866	0.023	0.009
CONTROL	DEN	0.006	0.093	0.513
CONTROL	DEN + RSV 50	0.016	0.058	0.189
CONTROL	DEN + RSV 75	0.031	0.014	0.005
CONTROL	DEN + RSV 100	0.513	0.014	0.005
DEN	DEN + RSV 50	0.065	0.513	0.419
DEN	DEN + RSV 75	0.014	0.072	0.006
DEN	DEN + RSV 100	0.005	0.028	0.006
DEN + RSV 50	DEN + RSV 75	0.339	0.221	0.018
DEN + RSV 50	DEN + RSV 100	0.018	0.044	0.009
DEN + RSV 75	DEN + RSV 100	0.095	0.074	0.072

DMSO: dimethyl sulfoxide; DEN: diethylnitrosamine; RSV: resveratrol

## Conclusion

The results of this study showed inhibition of DEN-induced HCC and a significant increase in apoptotic cancer cells at an RSV dose of 100 mg/kg. Therefore, RSV may be a promising treatment for HCC. Furthermore, because the number of human studies is still limited, further studies should be conducted to determine the most appropriate dose and duration of use of RSV.
